# Prevalence and risk factors of strongyloidiasis among schoolchildren in Sabach Sanjal and Upper Badibou districts in the North Bank East Region of The Gambia

**DOI:** 10.1016/j.parepi.2021.e00228

**Published:** 2021-10-21

**Authors:** Abdoulie M. Sanyang, Ebrima Joof, Alhagie Papa Sey, Sana Sambou, Zeehaida Mohamed, Bakary Sanneh

**Affiliations:** a National Public Health Laboratories, Ministry of Health, Bertil Herding High way, Kotu, the Gambia; b Epidemiology and Disease Control Unit, Ministry of Health, Bertil Herding Highway, Kotu, the Gambia; c School of Life Sciences, University of Nottingham, Nottingham, UK; d Department of Medical Microbiology & Parasitology, School of Medical Sciences, Universiti Sains Malaysia, 16150 Kubang Kerian, Kelantan, Malaysia

**Keywords:** Prevalence, Risk factors, S*trongyloides stercoralis*, Schoolchildren, Sabach Sanjal, Upper Badibou, The Gambia, NTDs, Neglected Tropical Diseases, WHO, World Health Organization, WHA, World Health Assembly, MDA, Mass Drug Administration, STHs, Soil-transmitted helminths, MRC, Medical Research Council, CLTS, Community-led total sanitation, LBS, Lower Basic School

## Abstract

**Background:**

Strongyloidiasis is a parasitic disease that mainly affects humans and is caused by a roundworm called *Strongyloides stercoralis*. It is endemic in humid tropical regions that include Africa, Latin America and Southern Asia. Among the public health important soil-transmitted helminths (STHs) classified as neglected tropical diseases, *S. stercoralis* is the most neglected. A study of schistosomiasis and STHs mapping was conducted and *S. stercoralis* larvae were detected using the utilized diagnostic method; thus, this current study described the prevalence and risk factors of *S. stercoralis* infection in districts of Sabach Sanjal and Upper Badibou in The Gambia.

**Methods:**

The cross-sectional study enrolled 851 schoolchildren, ages 7 to 14 years old. The participants were enrolled from 17 schools in Sabach Sanjal and Upper Badibou Districts. The WHO random sampling technique n/50 (25 boys and 25 girls) was used. Stool samples were collected from each participant and Kato-Katz smear method was used to screen for *S. stercoralis* infection.

**Results:**

Out of the total 851 pupils, 76 pupils (8.9%) were positive for *S. stercoralis* infection. The mean age of infected persons was 10.1 years (±2.2). The prevalence of infection was higher among females (9.2%) than males (8.7%). Rates of infection for age categories 7–10 years and 11–14 years were 12.4% and 4.2%, respectively. Rates of infection by districts were 12.3% for Sabach Sanjal and 7.1% for Upper Badibou. Schoolchildren from Sabach Sanjal were 1.6 times more likely to have strongyloidiasis compared to those from Upper Badibou (aOR = 1.64, *p*-value = 0.058). Schoolchildren aged 7–10 years were 3.2 times more likely to have strongyloidiasis infection compared to the 11–14-year-olds (aOR = 3.20, *p*-value <0.001). Schoolchildren who ‘sometimes’ have water or tissue after defaecation have more infection rate compared to those who ‘always’ have water or tissue after defaecation. However, this difference was not statistically significant (aOR = 1.36, p-value = 0.308).

**Conclusion:**

The study revealed the prevalence of strongyloidiasis in Sabach Sanjal and Upper Badibou districts of The Gambia. Kato-Katz technique might be inadequate for detecting *S. stercoralis*; thus, more studies are needed to determine the true prevalence of the disease in these two districts through the combined use of highly sensitive techniques such as Baermann, Koga Agar Culture and polymerase chain reaction.

## 1 Background

*Strongyloides stercoralis* is an intestinal nematode that causes a parasitic infection called strongyloidiasis in humans. It is endemic in humid tropical regions that include Africa, Latin America and Southern Asia (Puthiyakunnon et al., 2014; Abah and Arene, 2015). Among the public health important STHs classified as neglected tropical diseases (NTDs), *S. stercoralis* infection is the most overlooked and its prevalence is greatly underestimated (Shield et al., 2015; Wiria et al., 2015; Krolewiecki et al., 2010; Viney, 2017; Olsen et al., 2009). It is estimated that around 30–100 million people are infected in endemic areas globally, with most of them living in tropical and subtropical countries (Sharifdini et al., 2014; Jourdan et al., 2018; Schär et al., 2013). The disease is mainly associated with low socioeconomic level communities due to poor sanitation (Beknazarova et al., 2018).

*Strongyloides stercoralis* larvae are passed through the faeces of affected individuals and grow into the infective soil stage which infect people through skin penetration. The life cycle of *S. stercoralis* helps it to survive in the human host and contributes to chronic diseases that can exist for 75 years (Kwit et al., 2015; Khieu et al., 2014a; Verhagen et al., 2013; Prendki et al., 2011). *S. stercoralis* is auto infective and so unless diagnosed and treated, most infected individuals remain so for life. The existence of chronic disease increases with age as the opportunities for exposure increase. Clinical manifestations of infection include weight loss, malnutrition, diarrhoea, skin rashes and blocked bowel. The morbidity of strongyloidiasis may lead to anaemia, poor cognitive development, nutritional deficiency, and poor growth, affecting children's educational performance (Moore et al., 2015). A fatal systemic infection can occasionally occur in some infected persons (Puthiyakunnon et al., 2014). The most common risk factors are immunosuppression triggered by corticosteroids and infection with human T-lymphotropic virus (Nematian et al., 2008). The risk of developing strongyloidiasis is high for adolescents, including children of school age. Children are usually affected by parasitic infections and malnutrition (Paltridge and Traves, 2018). Strongyloidiasis transmission is facilitated by practices such as walking barefooted, playing without shoes, consuming fruits and vegetables which are not well washed by children (WHO, 2002; Zeehaida et al., 2011). Besides, poor personal hygiene, occupational exposure to soil or sand, farm animals, overcrowding, inadequate water supply and open defaecation contribute to dissemination of infection (Schär et al., 2013; Kola et al., 2013; CDC, 2013; WHO, 2018; Beknazarova et al., 2016; Schär et al., 2016; Boggild et al., 2016). Strongyloidiasis prevalence is higher in men compared to women (Alemu et al., 2017; Gudina et al., 2018; Terefe et al., 2019), as well as in farmers (Alemu et al., 2017; Gudina et al., 2018; Al-Mekhlafi et al., 2019) and in rural settings than in urban settings (Abera et al., 2013). Due to the high prevalence of the disease burden in children, the WHO aims to treat 75% of schoolchildren at risk of helminthiasis through chemotherapy (Paltridge and Traves, 2018).

Baseline data on the prevalence and risk factors of *Strongyloides stercoralis* infections are imperative for public health interventions because this helminth does not respond well to standard albendazole/mebendazole but needs ivermectin for elimination (Echazú et al., 2017; Datry et al., 1994). Global prevalence and morbidity of strongyloidiasis are commonly underestimated (Krolewiecki et al., 2013) and The Gambia is no exception. To date, there is limited data on this parasitic infection among schoolchildren in The Gambia. A review of health facility records did not show *S. stercoralis* prevalence in the country (unpublished data). After the London Declaration of 30th January 2012, which called for eliminating NTDs in some countries, the drive to control schistosomiasis and other NTDs has gained traction (World Health Organization, 2021; World Health Organization, 2012). This call (2012 WHA Resolution 65.21) increased efforts globally to control disease morbidity to achieve the elimination goal. The preferred control for strongyloidiasis is preventive chemotherapy with ivermectin. Before implementing massive drug administration (MDA) campaigns, an evaluation of infection prevalence must be performed. Typically, this is usually done using conventional parasitological methods (O'Connell and Nutman, 2016). Definitive diagnosis depends on larvae presentation and identification in stool specimens (Requena-Méndez et al., 2013). Kato-Katz technique is recommended for field estimation of STHs infection and schistosomiasis because it provides a standardised reading, glycerin enhances microscopy and the microscopists can be easily trained (Katz et al., 1972). On the other hand, Kato-Katz technique was found not suitable for *S. stercoralis* (Machicado et al., 2012; Hailu et al., 2020; WHO, 2020); however, during the field estimation, some of *S. stercoralis* may be recovered by this technique. The prevalence of strongyloidiasis by Kato-Katz method among preschool and schoolchildren was reported at 4% in Ethiopia (Amare et al., 2021), 2.1% in Madagascar (Hakami et al., 2019), 2.2% in Zanzibar (Knopp et al., 2008a), 9.7% in the Eastern Caribbean Sea (Kurup and Hunjan, 2010), 5.8% in Ethiopia (Legesse and Erko, 2004), 0.2% in Angola (Mirante et al., 2016) and 2.7% in Thailand (Sedionoto et al., 2019).

To reduce the burden of strongyloidiasis, the strategy to control STHs should include *S. stercoralis* (Krolewiecki et al., 2013). The Gambia has an NTD programme aimed at mapping and eliminating schistosomiasis and STHs, but does not include strongyloidiasis. Given the widespread prevalence of *S. stercoralis* infection and its ability to cause morbidity and mortality especially in resource-constrained countries (Keiser and Nutman, 2004), we decided to assess its prevalence alongside the targeted STHs during the 2015 NTD mapping survey. The study used this NTD mapping survey to gain information on the status of *S. stercoralis* infection.

## 2 Methods

### 2.1 Study setting

The study was conducted in Sabach Sanjal and Upper Badibou districts located in the eastern part of the North Bank Region of The Gambia. Sabach Sanjal is located on latitude 13 35′ 0” N and longitude 15 26′ 0” W while Upper Badibou is on latitude 13 34′ 25” N and longitude 15 35′ 55” W. Both districts have the river Gambia at their southern part and borders with Senegal at their northern part. Sabach Sanjal is about 125 km away from the capital city, Banjul. The two districts have an estimated population of 92, 359 (https://www.gbosdata.org/downloads/census-2013-8, n.d.), and their main occupations are farming (crop cultivation, cattle rearing, and fishing) and business.

### 2.2 Study design and population

The survey was a cross-sectional study that recruited schoolchildren. It was conducted as part of the 2015 schistosomiasis and STHs mapping survey. While stool samples were examined for *S. mansoni* and the three major STHs infections, *S. stercoralis* was also observed and recorded. The NTD mapping protocol recommended by the World Health Organization was used to determine the sample size. The WHO random sampling technique follows the WHO guide for mapping schistosomiasis in the African region which recommends randomly selecting 50 schoolchildren (25 boys, 25 girls) per school/village. The *n* = 50 is the total number of children sampled per school/village, and the recommended age group is 7–14 years. Based on most similar studies, age was categorised into two subgroups of 7 to 10 years and 11 to 14 years during the analysis in order to determine which group are at higher risk for strongyloidiasis.

**Fig. 1:**
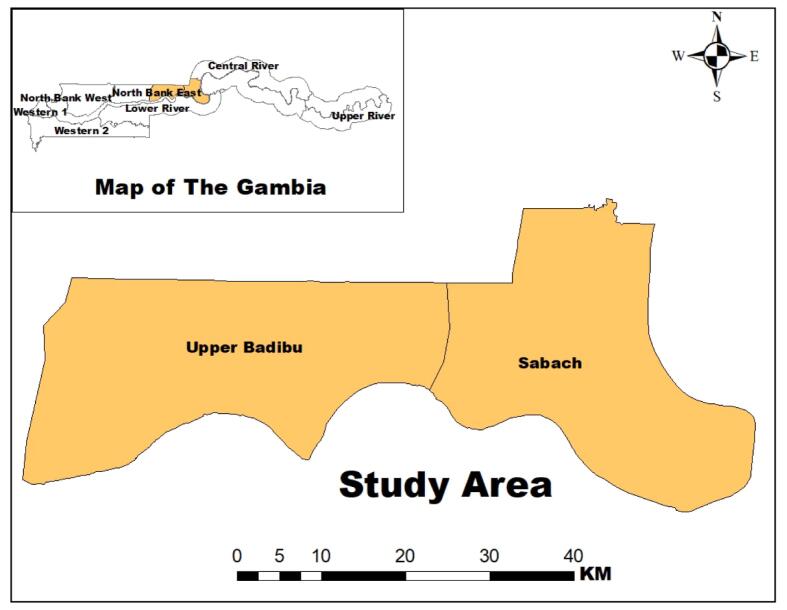
A map of the two districts where the study was carried out.

**Fig. 2:**
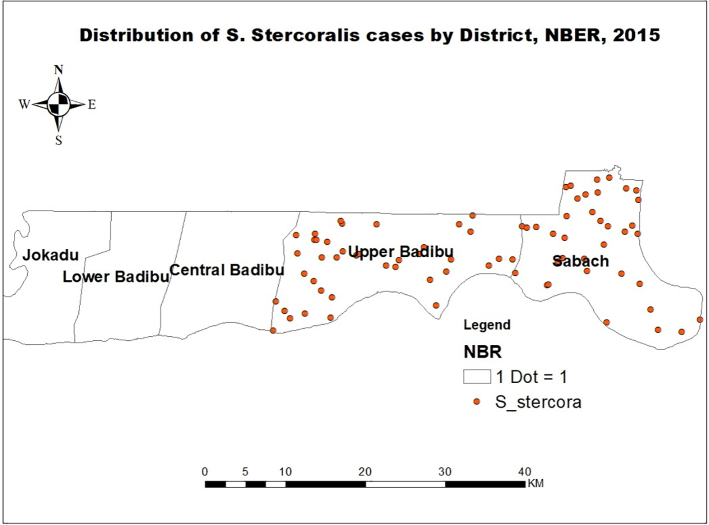
Distribution of *Strongyloides stercoralis* cases in the study districts.

**Fig. 3:**
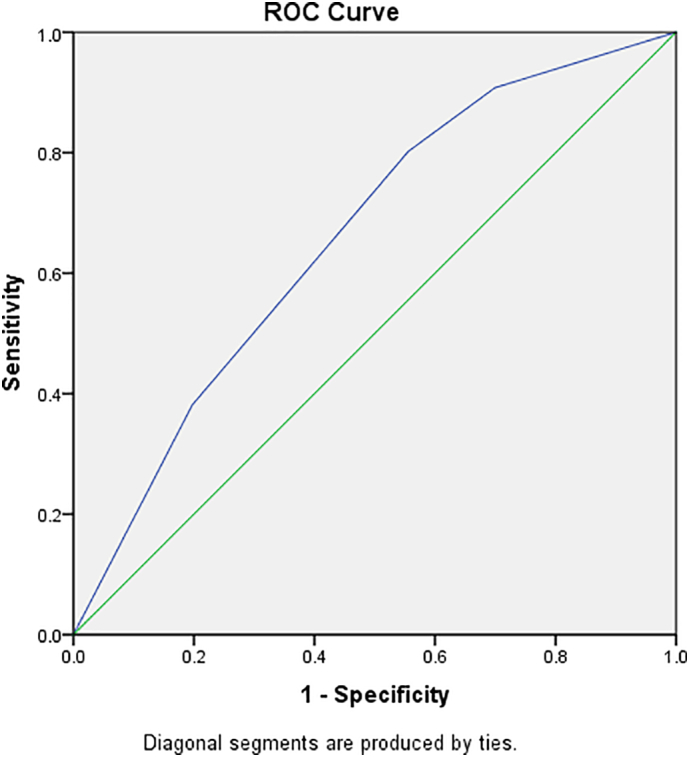
ROC curves derived from the multiple logistic regression final model with district and age.

The study was conducted in two districts in the North Bank East Region (NBER) (Fig. 1). Approximately 50 students between the ages of 7 and 14 years (25 boys and 25 girls) were randomly selected from each of 6 schools in Sabach Sanjal and 11 schools in Upper Badibou. In Sabach Sanjal, 51 participants (25 boys and 26 girls) were enrolled in one school, and from the remaining 5 schools, 50 participants were enrolled in each giving a total of 301. In Upper Badibou, 50 participants were enrolled in each of the 11 selected schools giving a total of 550 participants. In total, 851 participants from 17 schools were enrolled in the study conducted between May and June 2015. (See Fig. 2, Fig. 3.)

### 2.3 Inclusion and exclusion criteria

Schoolchildren aged 7 and 14 and residing in the districts were selected for the study. Participants' parents gave consent for their children to be recruited in the study. All participants with complete samples and data were included. Exclusion criteria includes students who were absent or sick during the study period.

### 2.4 Sample collection

Pre-labelled screw-capped plastic stool containers (bearing barcode and date of sample collection), tissue and wooden spatula were given to the participants to produce stool samples. Each participant was escorted to the toilet by a member of the research team and asked to provide a three-quarter filled container of stool. The samples were obtained between 8:00 am and 12:00 noon.

### 2.5 Laboratory procedures

The stool samples were processed at ambient temperature and transported within 2 h of collection to a regional laboratory at Farafenni District Hospital. Samples were analysed using Kato-Katz method (Katz et al., 1972). For each sample, spatula was used to collect portion of the stool sample and placed on a tissue paper. After this, a screen was used to press on top of the sample. This allows the sample to sieve through the gauze and accumulate above the gauze. The spatula was used to scrape the sieved sample and transfer about 0.0147 g of the sieved sample to a hole of 6 mm on a 1.5 mm thick template placed on a microscope slide. The template was then diligently removed leaving the sample on the slide intact. The pre-soaked cellophane strip was used to cover the faecal material and the slide was inverted and pressed firmly on a smooth hard surface. The slide with the cellophane was placed upwards to allow water to evaporate while glycerol cleared the faeces. The prepared Kato-Katz slides were then examined under a light microscope for *S. stercoralis* larvae. Two independent trained microscopists with over 10 years of experience examined the slides. The microscopy results were recorded on a laboratory reporting sheet.

### 2.6 Laboratory results

Parents of schoolchildren with positive results were notified, and treatments were prescribed by a doctor. The researchers gave an awareness of talk/health education on preventing strongyloidiasis in schoolchildren.

Field survey data was collected using the Task Force for Global Health (Atlanta, USA) LINKS ® data collection system. This is an android phone-based programme with an in-built questionnaire that helps the various teams to standardise epidemiological data collection. The questions asked were on water and latrine use practices including source of drinking water in the school and at home, type of water source, latrine availability in school and type, and conditions of existing latrine. The teams collected the GPS coordinates, the total number of pupils, the total number of boys and girls in school, toilet availability, water supply and handwashing facilities at the school level. All the survey data were managed in the LINKS system. Study participants' age and gender were also entered in the in-built LINKS system, while laboratory results on *S. stercoralis* infection were recorded on lab forms. Raw data were downloaded and then curated from LINKS framework server into Microsoft Excel spreadsheets (Microsoft Corporation, Redmond, WA, USA) for analysis (Sedionoto et al., 2019).

### 2.7 Data analysis

A total of 851 participants were registered in the study, all of whom had complete data and were included in the final analysis. The statistical analysis conducted for this study were descriptive statistics and binary logistic regression analysis. The descriptive analysis focused on mean, frequencies, percentages, and standard deviation. For binary logistic regression, simple analysis was performed first to screen for important variables (*p*-value <0.25), and then multiple logistic regression analysis was performed to obtained adjusted Odds Ratios (aOR). Analysis of logistic regression was used to classify the significant factors associated with the outcome of interest, *S. stercoralis* infection. Adjusted odds ratios (aOR) with 95% Cl were estimated to assess the strength of associations and statistical significance. All statistical analyses were carried out using SPSS version 26. *S. stercoralis* infection distribution maps were created using Health Mapper version 4.3.2 (WHO Public Health Information and Geographic Information Systems, Geneva, Switzerland).

### 2.8 Ethical issues

The Scientific Coordinating Committee of the Joint Gambia Government and MRC Ethics Committee reviewed and approved the study (document no: SCC 1415). School head teachers were informed and before the commencement of the study, parents of the participating children gave consent on behalf of their children.

## 3 Results

### 3.1 Socio-demographic characteristics of the study participants

The study enrolled a total of 851 schoolchildren and all of them completed the survey (Table 1). They comprised of 425 boys (49.9%) and 426 girls (50.1%) between the age bracket of 7–14 years from the two study districts (Table 2). The mean (± 2SD) age of infected individuals was 10.1 years (±2.2) (Table 2). Participants' ages were grouped into 7–10 years and 11–14 years.

**Table 1:** Prevalence of *Strongyloides stercoralis* parasite among the seventeen schools.

Schools	No examined	No infected	Prevalence (%)
Sabach Sanjal district			
Sinchu Njabo LBS	50	0	0
Challa LBS	51	0	0
Kataba LBS	50	8	16
Kunjata LBS	50	15	30
Loumen LBS	50	14	28
Pallen Fula LBS	50	0	0

Upper Badibou district			
Ballingho LBS	50	7	14
Maka Ferafenni LBS	50	27	54
Kerr Biram LBS	50	1	2
Ngerr LBS	50	0	0
St. John the Baptist LBS	50	0	0
Yallal Ba LBS	50	4	8
Konteh kunda LBS	50	0	0
Jajari LBS	50	0	0
Katchang	50	0	0
Kerr Ndongo LBS	50	0	0
Yallal tankonjala LBS	50	0	0
Sinchu Njabo LBS	50	0	0
Total	851	76	9

LBS = Lower Basic School.

**Table 2:** Demographic information and risk factors of the participants.

Variables	Frequency	Percentage
District		
Sabach sanjal	301	35.4
Upper Badibou	550	64.6
Gender		
Male	425	49.9
Female	426	50.1
Age group		
7–10 years	492	57.8
11–14 years	359	42.2
Latrine in School		
Yes	851	100
No	0	0
Available water or tissue in the toilet		
Always	701	82.4
Sometimes	150	17.6
Latrine type		
Without slab	151	17. 7
With slab	200	23.5
Ventilated Improved Pit latrine	400	47
Latrine condition		
Poor	50	5.9
Fair	50	5.9
Good	601	70.6
Excellent	150	17.6
Availability of drinking water in the schools		
Yes	851	100
No	0	0
Provision of handwashing after defaecation		
Yes	851	100
No	0	0
Facility available after defaecation		
Water only	150	17.6
Water and soap	701	82.4

### 3.2 Prevalence and risk factors of strongyloidiasis

Out of the total 851 pupils (Sabach Sanjal: 301, Upper Badibou: 550), 76 pupils (8.9%) were positive for *S. stercoralis*. Of the positive cases, 37 (49%) were from Sabach Sanjal and 39 (51%) from Upper Badibou (Table 3). The prevalence of infection was higher among females (9.2%) than males (8.7%). The prevalence by age category was 12.4% among 7–10 years category and 4.2% among 11–14 years category (Table 3). The prevalence by districts was 12.3% in Sabach Sanjal and 7.1% in Upper Badibou (Table 3). Table 1 shows the prevalence of *S. stercoralis* infection among the 17 schools. More than half (54.0%) of the tested population in Maka Farafenni school had positive stool samples, thus constituting the school with the highest disease burden in the two districts. Meanwhile, Kerr Biram School recorded the lowest prevalence of 2.0% (Table 1). In terms of risk factors of infection, schoolchildren from Sabach Sanjal were 1.6 times more likely to have *S. stercoralis* infection compared to those from Upper Badibou (aOR = 1.64, *p*-value = 0.058). Schoolchildren aged 7–10 years were 3.2 times more likely to have *S. stercoralis* infection compared to the 11–14-year olds (aOR = 3.2, *p*-value <0.001) (Table 3). Schoolchildren who sometimes have water or tissue after defaecation have more infection rate compared to those who always have water or tissue after defaecation. However, this difference was not statistically significant (aOR = 1.36, p-value = 0.308). No significant associations were found between *S. stercoralis* infection and variables such as gender, latrine in school, availability of drinking water in school, latrine type, latrine condition, provision of handwashing material after defaecation and facility available after defecation (p-value >0.05) (Table 3). Association of district and age group interaction was also assessed in the regression model and found not to be significant (p-value = 0.529). Apart from *S. stercoralis,* 4.3% and 2.5% of the studied children were found infected with schistosomiasis and STH, respectively.

**Table 3:** Factors associated with test positivity of strongyloidiasis among school children in the two districts of The Gambia, 2015.

Factors	Negative (%)	Positive (%)	COR (95% CI)	p-value	AOR (95% CI)	p-value
District						
Sabach Sanjal	264 (87.7)	37 (12.3)	1.84 (1.14, 2.95)	0.012	1.64 (0.98, 2.73)	0.058
Upper Badibou	511 (92.9)	39 (7.1)	1			
Gender						
Male	388 (91.3)	37 (8.7)	1			
Female	387 (90.8)	39 (9.2)	1.06 (0.66, 1.69)	0.818		
Age group						
7–10 years	431 (87.6)	61 (12.4)	3.25 (1.81, 5.81)	<0.001	3.20 (1.79, 5.74)	0.001
11–14 years	344 (95.8)	15 (4.2)	1			
Latrine in School						
Yes	774 (91.1)	76 (8.9)	–			
No	0 (0)	0 (0)	–			
Availability of water or tissue for use after defaecation					1.36 (0.75, 2.48)	0.308
Always	644 (91.9)	57 (8.1)	1			
Sometimes	131 (87.3)	19 (12.7)	1.64 (0.94, 2.85)	0.08		
Latrine type						
Without slap	151 (100)	0 (0)	–			
With slap	270 (90.0)	30 (10.0)	–			
VIP	354 (88.5)	46 (11.5)	–			
Latrine condition						
Fair	50 (100)	0 (0)	–			
Moderate	50 (100)	0 (0)	–			
Good	539 (89.7)	62 (10.3)	–			
Excellent	136 (90.7)	14 (9.3)	–			
Availability of drinking water in the school						
Yes	775 (91.1)	76 (8.9)	–			
No	0 (0)	0 (0)	–			
Provision of handwashing after defaecation						
Factors	Negative (%)	Positive (%)	COR (95% CI)	p-value	AOR (95% CI)	p-value
Yes	775 (91.1)	76 (8.9)	–			
No	0 (0)	0 (0)	–			
Facility available after defaecation						
Water only	135 (90.0)	15 (10.0)	1			
Water & Soap	640 (91.3)	61 (8.7)	0.86 (0.47, 1.56)	0.613		

COR = crude odds ratio, AOR = adjusted odds ratio, Cl = confidence interval.

## 4 Discussion

The move to control schistosomiasis and other NTDs has gained momentum after the London Declaration of 30th January 2012, which had advocated for the elimination of NTDs including *Strongyloides stercoralis* in some countries (Abah and Arene, 2015; Wiria et al., 2015). This call (2012 WHA Resolution 65.21) has intensified global efforts to reduce the disease burden and achieve elimination targets. The distribution of *S. stercoralis* infection worldwide varies enormously from one country to another and even within the same country, depending on socio-economic and ecological conditions (Olsen et al., 2009; Schär et al., 2013; Ngui et al., 2016). To our knowledge, this is the first school-based survey in The Gambia that assessed the prevalence of *S. stercoralis* infection using a traditional parasitological technique, Kato Katz. The study showed that 8.9% of the schoolchildren had *S. stercoralis* infection. This value is higher compared to the prevalence of *S. stercoralis* infection found in other countries such as 2.1% in Madagascar (Hakami et al., 2019), 2.2% in Zanzibar (Knopp et al., 2008a), 5.8% in Ethiopia (Legesse and Erko, 2004), 0.2% in Angola (Mirante et al., 2016) and 2.7% in Thailand (Sedionoto et al., 2019). However, our prevalence is comparable to the 9.7% prevalence reported in the Eastern Caribbean Sea (Kurup and Hunjan, 2010) and the 10.8% prevalence in Zanzibar (Knopp et al., 2008b). The prevalence is lower compared to studies from Malaysia, Ethiopia, Angola, Cambodia, India, Nigeria and Cote d'Ivoire, where considerable higher prevalence of 15.8%, 20.7%, 21.4%, 12.8%, 31.6%, 36%, 13.8%, 4–48% respectively were reported (Amor et al., 2016; Dacal et al., 2018; Forrer et al., 2017; Maikenti et al., 2020; Rajkumari and Jayaseelam, 2016; Glinz et al., 2010). However, the use of different techniques makes a comparison of findings difficult. Apart from that, the studied population/group, the different culture, type of food eaten, personal hygiene and sanitary facilities of each country would also influence the study outcome.

There was striking differences in the rate of infection between the various schools in this study. The study results showed that the prevalence of strongyloidiasis was higher in Sabach Sanjal than in Upper Badibou district. Upper Badibou is slightly more developed than Sabach Sanjal. In majority, the occupation of people in Sabach Sanjal is farming and they often miss routine reproductive child clinics and vaccination campaign including deworming activities, while people in Upper Badibou do business and partly engage in some farming activities. The study also found comparable prevalence of strongyloidiasis between males and females. A previous study found that females were more infected with strongyloidiasis than their male counterparts (Panda et al., 2012). Other studies, however, found the contrary that males were more infected with *S. stercoralis* than females (Kola et al., 2013; Terefe et al., 2019; Obeta, 2019; Khieu et al., 2014b). This difference in infection rate between males and females could be attributed to the socioeconomic and cultural factors that exist in the study settings. For example in Cambodia, it is attributed to the main economic activity that is subsistence-level rice farming (Khieu et al., 2014b).

This current study found that *S. stercoralis* infection had significant association with only age group and district out of all the potential risk factors examined. Age group 7–10 years were 3.2 times more likely to develop strongyloidiasis compared with the age group 11–14 years. This is consistent with previous observations by Khieu and other (Khieu et al., 2014b). and Agbo and colleagues (Oche Agbo et al., 2019) who found that age is a risk factor for strongyloidiasis. In the study by Agbo and colleagues, children of age group 8–12 years in Katsina, Nigeria has significantly higher risk of infection due to walking bare footed, playing with sand, eating food without washing hands and personal hygiene. However, in our study, we found no correlation between *S. stercoralis* infection and the availability of facilities after defaecation as well as water or tissue availability. This, however, contrasts with findings of Schär et al. (Schär et al., 2013) who found an association between the absence of sanitary facilities and strongyloidiasis.

Concerning the two districts, we observed significant heterogeneity between the schools; three schools in the Sabach Sanjal district and four schools in the Upper Badibou district had *S. stercoralis* infection. The highest and lowest prevalence were both found in Upper Badibou district. We could not distinguish any differences between the participants of the two districts or schools in the demographic data, latrine facilities or personal hygiene conditions. Therefore, we are unable to determine why participants from certain schools had higher infection rates. A recent assessment report conducted by the environment unit of the Ministry of Health, The Gambia found that most communities in both districts lack access to potable drinking water and eleven communities are still practicing open defaecation (CLTS, 2019). However, in this study, the detection of *S. stercoralis* larvae is part of a bigger schistosomiasis and STHs mapping which uses an AFRO questionnaire capturing data on schistosomiasis and STHs, thus the environmental variables and other potential risk factors for *S. stercoralis* such as walking barefooted, open defaecation, playing without shoes and eating fruits and vegetables that are not well washed within the two districts' school climate were not evaluated. Previous studies have reported that *S. stercoralis* infection is correlated with temperature, rainfall, open defaecation, inadequate portable water, farming, walking barefooted, playing without shoes, consuming fruits and vegetables which are not well washed (Zeehaida et al., 2011; Kola et al., 2013; CDC, 2013; Beknazarova et al., 2016; Forrer et al., 2019). Differences between the two districts in socioeconomic demography, environmental factors and children's activities may explain our observations.

The study revealed a significant prevalence of *S. stercoralis* among schoolchildren in Sabach Sanjal and Upper Badibou districts. However, the study utilized only Kato-Katz method to detect *S. stercoralis* larvae in stool samples. More suitable approaches to detect *S. stercoralis* larvae such as larva recovery method, agar plate culture and polymerase chain reaction could be used to increase the detection rate. For example, the examination of multiple stool samples per participant coupled with the use of a combination of more sensitive techniques such as Baermann method and Koga agar plate can increase the sensitivity up to 70% or 93% (Khieu et al., 2013). The prevalence of the disease could have been higher in these two districts of North Bank East Region if more sensitive diagnostic techniques were employed in the present study as Kato-Katz is not a suitable technique for detection and diagnosis of *S. stercoralis* (WHO, 2020). A study that compared Kato-Katz and APC among Peruvian Amazon communities found out infection rate of 0% with Kato-Katz technique and 22% with APC (Machicado et al., 2012). On the other hand, a prevalence of 24.4% among schoolchildren in Cambodia was obtained using combined two methods, Baermann and Koga agar plate culture techniques (Khieu et al., 2013). Additionally, a recent systematic review of 82 studies in Africa found Kato-Katz to be a less popular tool for diagnosis of *S. stercoralis* with just 2 of these studies utilising the technique for detection of *S. stercoralis* (Hailu et al., 2020). The study also found that Kato-Katz detected *S. stercoralis* larvae in only 8 out of 810 participants (Hailu et al., 2020). These are further evidence that *S. stercoralis* is not an ideal method for diagnosis of *S. stercoralis*. Given the evidence highlighted by the previous studies, our findings may be an underestimation of the true prevalence estimates of *S. stercoralis* in the study area. Further studies that will employ more suitable and sensitive techniques such as Baermann method, Koga agar plate and polymerase chain reaction are required to elucidate the true status and prevalence of *S. stercoralis* in Upper Badibou and Sabach Sanjal. Despite the low prevalence, our study revealed the occurrence of *S. stercoralis* in Sabach sanjal and upper Badibou for the first time.

Our study examined double Kato-Katz slides prepared from a single stool sample per participant to maximise the *S. stercoralis* larvae's excretion in stool specimens. This is part of a strategy to increase the yield of larvae. Another study revealed that larva output in faecal specimens ranged from 0.003 larvae per gram to 151.2 larvae per gram (Khieu et al., 2013). However, the use of a Kato-Katz technique only in this study is a limitation and cannot efficiently detect all *S. stercoralis* infection (Machicado et al., 2012; Hailu et al., 2020; WHO, 2020). Thus, the result of our study should be interpreted cautiously. Questionnaires that capture more relevant risk factors such as walking barefooted, contact with the soil, contact with human waste or sewage, contact with farm animals, eating unwashed fruits and vegetables, farmer's children, awareness about worm infections and open defaecation should also be used.

We conducted awareness campaigns on the prevention of the disease and treated the affected schoolchildren with albendazole, a widely available antihelminth. Another treatment option is ivermectin which is superior in treating strongyloidiasis than albendazole. The effectiveness of ivermectin is documented in many studies and its cure rate is better than albendazole (Henriquez-Camacho et al., 2016).

## 5 Conclusion

The study revealed notable prevalence of strongyloidiasis in Sabach Sanjal and Upper Badibou districts of The Gambia. Kato-Katz technique might be inadequate for detecting *S. stercoralis*; thus, more studies are needed to determine the true prevalence of the disease in these two districts through the combined use of highly sensitive techniques such as Baermann, Koga Agar Culture and polymerase chain reaction.

The following are the supplementary data related to this article.

Supplementary material 1 Additional file 1. Standard Operating Procedure for Kato-Katz technique. Supplementary material 1

Supplementary material 2 Additional file 2. *Strongyloides stercoralis* Positive data. Supplementary material 2

Supplementary material 3 Additional file 3. *Strongyloides stercoralis* Negative data. Supplementary material 3

Supplementary material 4 Additional file 4. *Strongyloides stercoralis* Risk factors data. Supplementary material 4

Supplementary material 5 Additional file 5. Risk factors questionnaire. Questions about different toilet activities and other related sanitary facilities and their responses. Supplementary material 5

## Competing interests

The authors declare that they have no competing interests.

## Funding

This study was not funded because it was conducted as an axillary project alongside another study (field evaluation of a schistosomiasis rapid test kit) funded by The Taskforce for Global Health. Therefore, the researchers acknowledge the support of the Taskforce for Global Health (A992A78-CC70–4846-AF50BE65573CA71B).

## Authors' contributions

AMS, EJ, APS, SS and BS conceived and designed the study. AMS, SS and BS trained the personnel on study protocols and conducted field supervision. EJ and APS conducted field and laboratory work. AMS and APS carried out statistical analysis and AMS drafted the paper. All aspects of the manuscript were reviewed critically by ZM, EJ and BS. The final version of the manuscript was read and accepted by all authors.

## Declarations

Availability of data and materials

All the data used in this study are included in a separate supplementary document submitted along with this manuscript and can be obtained from the corresponding author on a reasonable request.

Ethics approval and consent to participate

The Scientific Coordinating Committee and the Gambia Government and Medical Research Council Joint Ethics Committee provided ethical clearance for the study (document no: SCC 1415). School head teachers were informed and before the commencement of the study, they obtained permission from students' parents, guardians or wards. On behalf of all students whose parents or guardians have consented, the headmaster/mistress signed written consent. Furthermore, informed consent was obtained from each study participant as the data collection teams enrolled them.

Consent for publication

Not applicable.

Competing interests

The authors declare that they have no competing interests.

Author details

^1^ National Public Health Laboratories, Ministry of Health, Kotu, The Gambia. ^2^Epidemiology and Disease Control Programme, Ministry of Health, Kotu, The Gambia.

^1,3^School of Life Sciences, University of Nottingham, Nottingham, UK.

^4^Department of Medical Microbiology & Parasitology, School of Medical Sciences, Universiti Sains Malaysia, 16,150 Kubang Kerian, Kelantan, Malaysia.
