# Supplementary material for: Prevalence and risk factors of strongyloidiasis among schoolchildren in Sabach Sanjal and Upper Badibou districts in the North Bank East Region of The Gambia
Source: Parasite Epidemiol Control. 2021 Oct 21;15:e00228. doi: 10.1016/j.parepi.2021.e00228 (PMC8716566; doi:10.1016/j.parepi.2021.e00228)
Supplement: Supplementary material 1 — Additional file 1. Standard Operating Procedure for Kato-Katz technique. [file mmc1.docx]

Epidemiology and Disease Control Unit National Public Health Laboratories

**STANDARD OPERATING PROCEDURE FOR THE DETECTION OF** *Strongyloides stercoralis* **IN STOOL SAMPLES USING THE KATO-KATZ TECHNIQUE**

**1.0 Definition**

This SOP describes the procedure for preparing stool sample preparation and estimating worm load using the Kato-Katz technique.

**2.0 Purpose**

This SOP is to be used for microscopic screening of intestinal parasites (*Strongyloides stercoralis* eggs) during the study.

**3.0 Scope**

This SOP will be used by trained laboratory staff during the study to screen *Strongyloides stercoralis* eggs in human stool samples.

**4.0 Responsibility**

The laboratory technician/assistant should make sure that procedures are followed for the detection of eggs *Strongyloides stercoralis* using the kato-katz method.

**5.0 Safety**

It is the duty of the laboratory personnel to observe good laboratory practice when receiving, processing and analysing the samples at all times.

**Abbreviations:**

**SOP** stands for Standard Operating Procedure

**NTD** stands for Neglected Tropical Diseases

**WHO** stands for World Health Organization

**6.0 Materials and reagents**

- Applicator sticks (wooden/plastic)
- Screen, Stainless steel, nylon or plastic: 60-105 mesh
- Template, stainless steel, plastic, or cardboard.
- To ensure standardization of procedure, a hole of 6 mm on a 1.5 mm thick template was used to deliver 41.7 mg of faeces.
- Spatula (plastic)
- Microscope slides (25x75mm)
- Hydrophilic cellophane, 40-50um thick, strips 25 x 30 or 25 x 35 mm.
- Flat bottom jar with lid
- Forceps
- Toilet paper or absorbent tissue
- Newspaper/scrap paper
- Glycerol-malachite green or glycerol-methylene blue solution (1 ml of 3% aqueous malachite green or 3% methylene blue is added to 100 ml of glycerol and 100 ml of distilled water and mixed well). This solution is poured onto the cellophane strips in a jar and left for at least 24 hours prior to use.

1. **Procedure**

**7.0.1** Place a small mound of faecal material on newspaper or scrap paper and press the small screen on top so that some of the faeces are sieved through the screen and accumulate on top.

**7.0.2** Scrape the flat-sided spatula across the upper surface of the screen to collect the sieved faeces

**7.0.3** Place template with a hole on the centre of a microscope slide and add faeces from the spatula to fill the hole. Using the side of the spatula, pass over the template to remove excess faeces from the edge of the hole (the spatula and screen may be discarded or, if carefully washed, may be reused).

**7.0.4** Remove the template carefully so that the cylinder of faeces is left on the slide.

**7.0.5** Cover the faecal material with the pre-soaked cellophane strip. The strip must be very wet if the faeces are dry and less so if the faeces are soft (if excess glycerol solution is present on the upper surface of cellophane wipe with toilet paper). In dry climates, excess glycerol will retard but not prevent drying.

**7.0.6** Invert the microscope slide and firmly press the faecal sample against the hydrophilic cellophane strip on another microscope slide or a smooth hard surface such as a piece of tile or a flat stone. The faecal material will be spread evenly between the microscope slide and the cellophane strip. It should be possible to read newspaper print through the smear after clarification.

**7.0.7** Carefully remove the slide by gently sliding it sideways to avoid separating the cellophane strip or lifting it off. Next, place the slide on the bench with the cellophane upwards. Water evaporates while glycerol clears the faeces.

**7.0.8** For all except hookworm eggs, keep slide for one or more hours at ambient temperature to clear the faecal material prior to examination under the microscope. To speed up clearing and examination, the slide can be placed in a 40°C incubator or kept under direct sunlight for several minutes.

**7.0.9** *Ascaris* and *Trichuris* eggs will remain visible and recognisable for many months in these preparations. Hookworm eggs clear rapidly and will no longer be visible after 30-60 minutes. Schistosome eggs may be recognised for up to several months but it is preferable in an endemic schistosomiasis area; slide preparations are examined within 24 hours.

**7.1.0** The smear should be examined systematically and the number of eggs for each species reported. Later, multiply by the appropriate number to give the number of eggs per gram of faeces (by 24 for a 41.7 mg template).

**References:**

1. Basic Laboratory methods in medical parasitology, WHO, 1991.

**2. World Health Organization-**Action against worms (February, 2008).

.
