# Supplementary material for: Prevalence and risk factors of strongyloidiasis among schoolchildren in Sabach Sanjal and Upper Badibou districts in the North Bank East Region of The Gambia
Source: Parasite Epidemiol Control. 2021 Oct 21;15:e00228. doi: 10.1016/j.parepi.2021.e00228 (PMC8716566; doi:10.1016/j.parepi.2021.e00228)
Supplement: Supplementary material 4 — Additional file 4. Strongyloides stercoralis Risk factors data. [file mmc4.docx]

**Gambia AFRO School WASH - Schisto STH v2 (eng)**

| instanceID | Hidden from user |  |
| --- | --- | --- |
| Q1_Recorder | Enter Recorder ID | User entered integer |
| Q2a_School1 | Enter school code | User entered integer |
| Q2b_School2 | Enter school code again | User entered integer |
| Prompt1 | Ask teacher following questions | User entered text |

| **Variable Name** | **Question Text** | **Saved Value** |
| --- | --- | --- |
| meta Hidden from user | | |

| Q3_Water_source | Is there a source of drinking water in the school? | 1 Yes  2 No |
| --- | --- | --- |
| Q4_Water_where | Where is the source of drinking water? | 1 In the school  2 15 minutes or less round-trip from the school  3 More than 15 minutes round-trip from the school |
| Q5_Water_type | What type of water source? | 1 Unprotected spring  2 Protected spring  3 Unprotected dug well  4 Protected dug well  5 Hand pump/tubewell/borehole  6 Surface water (river, dam, lake, stream, canal)  7 Public piped water/tap/standpipe  8 Rainwater collection  9 Plastic bag water  10 Bottle water  11 Other |

Q5a_Water_other If other, specify User entered text

| Q6_Water_bodies | Are there accessible water bodies close to the school? | 1 Yes- 15 minutes of less round-trip  2 Yes - more than 15 minutes round-trip  3 No |
| --- | --- | --- |
|  |  |  |

| Q7_Latrine | Is there a latrine in the school? | 1 Yes  2 No |
| --- | --- | --- |
| Q7a_Latrine_water | Is there water or tissue for use after defecating? | 1 Always  2 Sometimes  3 Never |

Latrine Hidden from user

| Prompt2 Observe latrine User entered text | | | | | |
| --- | --- | --- | --- | --- | --- |
| Q8a_Latrine_type | What type of latrine is present? | 1 Pit latrine without slab or open pit  2 Pit latrine with slab  3 Ventilated improved pit latrine (VIP)  4 Flush or pour flush toilet  5 Other | | | |
| Q8b_Latrine_type_other If other, specify User entered text | | | | | |
| Q8c_Latrine_condition | What is the condition of the latrine? | 1 Poor (presence of flies, offensive odour and visible stool on floor, absence of roof/door)  2 Fair (presence of roof/door but dirty floor)  3 Moderate (clean, absence of roof/door)  4 Good (clean, odourless, no flies, presence of roof and door)  5 Excellent (Very clean, odourless, presence of door, roof, and availability of water) | | | |
| Q8d_Latrine_water | Is there water or tissue for use after defecating? | 1 Yes  2 No | | | |
| Q8e_Latrine_hand | Is there provision for hand washing after latrine use? | 1 Yes  2 No | | | |
| Q8f_Latrine_hand_type | What type of hand washing facilities are available? |  | | | |
|  |  |  | 1 No water  2 Water only  3 Water and soap | |  |
|  |  |  |  |  |  |

4 Water, soap, and non-disposable napkin

5 Water, soap, and disposable napkin

6 Other

| Q8g_Latrine_hand_other | If other, specify | User entered text |
| --- | --- | --- |
| Q9_Notes | Additional notes | User entered text |
| Start_time | Hidden from user | Timestamp of form open |
| End_time | Hidden from user | Timestamp of form save |
